# Comparison of Continuous and Programmed Intermittent Bolus Infusion of 0.2% Ropivacaine after Ultrasound-Guided Continuous Interscalene Brachial Plexus Block in Arthroscopic Shoulder Surgery

**DOI:** 10.1155/2022/2010224

**Published:** 2022-12-26

**Authors:** Hye-Jin Kim, Ji-Hye Baek, Seyeon Park, Ji-Uk Yoon, Gyeong-Jo Byeon, Sang-Wook Shin

**Affiliations:** ^1^Department of Anesthesia and Pain Medicine, Pusan National University Yangsan Hospital, Pusan National University School of Medicine, Yangsan, Republic of Korea; ^2^Research Institute for Convergence of Biomedical Science and Technology, Pusan National University Yangsan Hospital, Yangsan, Republic of Korea

## Abstract

**Background:**

Despite the clinical effectiveness of the programmed intermittent bolus (PIB) method for epidural analgesia, evidence for this method in continuous interscalene brachial plexus block (CIBPB) is unclear. This study aimed to investigate the pain relief effect after arthroscopic shoulder surgery according to the administration method by comparing the PIB and continuous infusion methods among the administration methods of local anesthetics.

**Methods:**

Sixty-four patients aged >19 years scheduled for elective arthroscopic shoulder surgery were enrolled and divided into two groups. Ultrasound-guided CIBPB was performed to control postoperative pain. The infusion pump was programmed so that 0.2% ropivacaine was continuously injected at 1.1 mL/h in group A, whereas in group B, 0.1 mL/h was continuously injected and 4 mL was periodically injected at 4 h intervals. In both groups, a further infusion of 4 mL of 0.2% ropivacaine was administered if the patient requested additional analgesia, and the lockout time was set at 30 min. Postoperative pain quality was assessed using a visual analog scale (VAS), and the incidence of patients requiring additional analgesics, motor blockade using a modified Bromage scale (MBS), and consumed doses of local anesthetic were assessed.

**Results:**

The VAS and incidence of rescue analgesics were performed when the patient could communicate voluntarily after admission to the post-anesthetic care unit, and at 24 and 48 h after surgery showed no significant difference between the two groups. The MBS at 24 h after surgery was significantly higher in group B (*p* = 0.038). In the comparison of consumed doses of local anesthetic, group B had a significantly higher bolus injection dose (*p* = 0.047) and frequency of bolus use in the 24 h after surgery (*p* = 0.034).

**Conclusion:**

The PIB method in CIBPB after arthroscopic shoulder surgery provided a similar analgesic effect, with a higher bolus injection dose of local anesthetic and increased motor blockade than the continuous infusion method.

## 1. Introduction

Arthroscopic shoulder surgery causes significant postoperative pain, and many analgesic methods can be used. In addition to systemic medications, various regional analgesic methods can be applied to control pain after arthroscopic shoulder surgery [1]. For effective postoperative analgesia, patient-controlled analgesia using continuous interscalene brachial plexus block (CIBPB) has been used [2].

Various factors have been considered to enhance the effectiveness of CIBPB. These factors include differences in the type of catheter, concentration and dose of local anesthetics, method of approach, and administration method [3]. The method of administration of local anesthetics may be continuously infused at a constant rate or intermittently injected at regular intervals. The main method of administering local anesthetics uses continuous infusion at a constant rate.

The programmed intermittent bolus (PIB) method differs from continuous infusion as the hourly block volume is given as a bolus rather than infused continuously. Such bolus administration is thought to result in a better spread of local anesthetic around the targeted nerves [4]. Several studies have described the improved efficacy of PIB epidural analgesia compared with continuous infusion epidural analgesia [5–8]. Despite the clinical effectiveness of the PIB method in epidural analgesia, evidence for this technique in regional anesthesia is unclear.

We hypothesized that the PIB technique would provide enhanced analgesia compared to a continuous infusion rate for CIBPB in patients undergoing arthroscopic shoulder surgery. This study aimed to investigate the pain relief effect after arthroscopic shoulder surgery according to the administration method by comparing the programmed intermittent bolus method and the continuous infusion method among the administration methods of local anesthetics.

## 2. Methods

### 2.1. Patient Enrollment

This study was approved by the Institutional Review Board of Pusan National University Yangsan Hospital (approval number: 05–2020-089), and the trial was registered with the Clinical Research Information Service (registration number: KCT0005197). After obtaining written informed consent, 64 patients aged >19 years with American Society of Anesthesiologists physical status I–II scheduled for elective arthroscopic shoulder surgery were enrolled. Patients who did not understand or could not participate in the study process, had a blood coagulation deficiency, neurological defects at the procedure site, allergic reaction to ropivacaine in previous surgery or procedures, or were pregnant women were excluded.

### 2.2. Randomization

At the preanesthetic visit, all patients were provided with a description of how to use a randomized allocation protocol and how to use a portable electronic injection pump before agreeing to participate. Random assignment to two groups was performed using a list of random numbers generated using Excel (Microsoft Corporation, Redmond, WA, USA). For a double-blind, randomized controlled study, the researcher who performed ultrasound-guided CIBPB could not measure the outcomes after surgery, and the outcome investigators were also blinded to the procedure.

### 2.3. Perioperative Management

When patients arrived in the operating room, they were placed in a lateral decubitus position with the operative shoulder free. After treating the skin with chlorhexidine alcohol and covering with a disinfectant cloth, the interscalene brachial plexus between the anterior and middle scalene muscle was identified using an ultrasonic 5.0–13.0 MHz linear probe (LOGIQ e; GE Healthcare, Princeton, NJ, USA). After infiltration of the needle insertion site with 4 mL of 2% lidocaine, a 10 cm 18-gauge Tuohy needle (NRFit PlexoLong Nanoline Kit; Pajunk GmbH, Geisingen, Germany) was inserted between the C-5 and C-6 nerve trunks and stimulated using an electrical nerve stimulator (Medipia ES400; Life-Tech, Stafford, TX, USA). The initial output of 1 mA, 2 Hz, and 0.2 ms was applied as the block needle advanced along the nerve trunks until upper arm muscle contractions were elicited, during which time the nerve stimulator was turned off. Before perineural catheter insertion, a loading dose of 10 mL 0.2% ropivacaine was administered. A 20-gauge perineural catheter (NRFit PlexoLong Nanoline Kit; Pajunk GmbH, Geisingen, Germany) was inserted through the needle and advanced to a depth of 1 cm beyond the needle tip between the C-5 and C-6 nerve trunks. After catheter placement, 10 mL of 0.2% ropivacaine was injected under ultrasound guidance to confirm that the local anesthetic diffused well around the nerves. The catheter was secured using a cutaneous adhesive suture with nylon 4-0 and attached to a chlorhexidine gluconate transparent securement dressing (Tegaderm CHG; 3M Corporation, St. Paul, MN, USA).

The success of the block was confirmed by the change in the cold sensation in the shoulder area caused by alcohol swabs within 15 min. The procedure was considered a failure when there was no change in sensation after 30 min.

General anesthesia was induced using 6 vol% desflurane. At the end of the surgery, 225 mL of 0.2% ropivacaine was infused through the indwelling catheter via a portable electronic injection pump (Accumate 1100; Woo Young Medical Co., Ltd., Jincheon, Chungbuk, Korea) for the first 48 h after surgery in both groups. In group A, the infusion pump was programmed so that 0.2% ropivacaine was continuously injected at 1.1 mL/h. If a patient felt pain, a further infusion of 4 mL of 0.2% ropivacaine was administered when the patient requested additional analgesia, and the lockout time was 30 min. In group B, the infusion pump was programmed with 4 mL of 0.2% ropivacaine periodically at 4 h intervals, continuously injected at 0.1 mL/h, and a patient requiring bolus dose of 4 mL with a lockout time of 30 min through the catheter. Both groups were set to receive the same amount of local anesthetic unless a patient requested additional analgesia.

### 2.4. Outcome Measurements

The primary outcome of this study was postoperative pain quality. Investigators who were blinded to the group assignments were assigned to assess postoperative pain quality using a VAS as well as the incidence of patients requiring additional analgesics, total consumed doses of local anesthetics, adverse events related to local anesthetics, and patient satisfaction regarding postoperative pain management. The VAS was recorded immediately after admission to the postanesthetic care unit and at 24 and 48 h postoperatively. When the VAS score was >60, and the patient wanted analgesics during the postoperative period, 30 mg of ketorolac or 20 mg of nefopam was injected. Nevertheless, if pain control was unsatisfactory, 50 *μ*g of fentanyl was administered. Additional analgesic requirements within 48 h after surgery were documented as the incidence of patients requiring additional analgesics by the investigators.

Motor blockade assessment was performed using the MBS for the upper extremities on a four-point scale (Grade 0: able to raise the extended arm to 90° for 2 s. Grade 1: able to flex the elbow and move the fingers but unable to raise the extended arm. Grade 2: unable to flex the elbow but able to move the fingers. Grade 3: inability to move the arm, elbow, or fingers) [9]. The MBS was also used when evaluating and recording the VAS.

Adverse events, including postoperative nausea and vomiting (PONV), dizziness, paresthesia, and urinary retention, were also evaluated. Patient satisfaction was assessed using a five-point scale, with 5 = very satisfied, 4 = satisfied, 3 = neutral, 2 = dissatisfied, and 1 = very dissatisfied.

After the administration of the drug through the patient-controlled device, the portable electronic injection pump was collected and connected to a computer to analyze the pump usage patterns (total dose, bolus injection dose, frequency of using bolus) of the subjects in the 48 h after surgery. Acculinker, version 1.0 (Woo Young Medical Co. Ltd., Jincheon, Chungbuk, Korea) was used for the analysis.

### 2.5. Statistical Analysis

Statistical analysis was performed using IBM SPSS Statistics for Windows, version 27.0 (IBM Corp., Armonk, NY, USA). The Student's *t*-test was used for demographic data such as age, height, weight, and anesthesia time, as well as the VAS score and total dose, bolus injection dose of local anesthetics, and frequency of using bolus administration. Sex and ASA physical status in demographic data, as well as the incidences of rescue analgesic administration, MBS, patient satisfaction with postoperative pain management, and incidences of adverse events, were compared using the *χ*^2^ test or Fisher's exact test. A statistical significance was set at a *p* value of <0.05.

### 2.6. Sample Size Estimation

This study was an experiment in which a local anesthetic was continuously injected using a portable electronic injection pump after an ultrasound-guided interscalene brachial plexus block was performed on a patient undergoing arthroscopic shoulder surgery. When the mean difference in the VAS scores measured 12 h after surgery was 15 or more, it was considered a clinically significant difference. In a previous study [10], the result of VAS measured 12 h after surgery in the intermittent injection group was 21.41 ± 18.37, and the type I (*α*) and type II (*β*) errors were measured with 0.05 and 0.2, respectively. The sample size for each group was 27. To increase the power of the test, five additional subjects were added to each group, and 32 subjects were selected for each group.

## 3. Results

**Figure 1:**
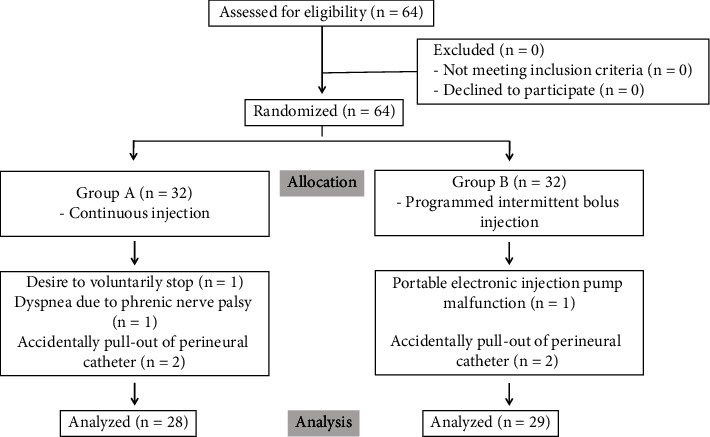
Patient enrollment and a study flowchart.

Sixty-four patients were initially included in the current study. Four patients in group A and three in group B did not complete the study. In group A, two patients had a dislodged perineural catheter during continuous brachial plexus block, one patient wanted to leave the study, and one patient wanted to leave the study due to dyspnea caused by phrenic nerve palsy. The perineural catheter was removed in the patients, and they underwent general anesthesia as scheduled, recovering to normal after follow-up. In group B, two patients were unable to participate further due to dislodging of the perineural catheter and one patient found that the local anesthetic was not properly injected through the catheters due to device malfunction in the ward, and no further evaluations were conducted after that. These seven patients were administered opioid-based intravenous patient-controlled analgesia for postoperative pain management (Figure 1). There were no differences in the demographic data between the two groups (Table 1).

The VAS when the patients could communicate voluntarily after admission to the PACU, at 24 and 48 h after surgery showed no significant difference between the two groups. There was no difference in the incidence of rescue analgesics administered immediately after admission to the PACU, at 24 and 48 h after surgery in the two groups (Table 2).

The MBS was used to evaluate motor blockade in the two groups, with group B having significantly higher MBS at 24 h after surgery (*p* = 0.038, Table 3).

After the administration of local anesthetic through the patient-controlled device, the portable electronic injection pump was collected and connected to a computer to analyze the pump usage patterns (total dose, total bolus injection dose, and frequency of bolus injection) of the patients for 48 h after surgery. There was no difference in the total dose and total bolus injection dose of local anesthetics in the two groups. However, group B had a significantly higher bolus injection dose (*p* = 0.047) and frequency of bolus use in the 24 h after surgery than group A (*p* = 0.034, Table 4).

There was no significant difference in patient satisfaction between the two groups (Table 5). Adverse events including PONV, dizziness, hypotension, paresthesia, and urinary retention were evaluated. There was no significant difference in the incidence of adverse events between the two groups (Table 6).

## 4. Discussion

The PIB method of local anesthetics has shown greater efficacy in providing epidural analgesia compared to continuous injection methods in several studies [5, 11–13]. Likewise, the efficacy of the PIB method in continuous peripheral nerve blocks have also been reported in the following studies. In a study comparing the PIB and continuous infusion methods with the popliteal sciatic catheter in patients undergoing hallux valgus correction surgery, the PIB method resulted in a local anesthetic-sparing effect. Both methods showed equivalent analgesic effects, and patients using the PIB method were administered fewer boluses, resulting in a lower total consumption of local anesthetics [14]. These studies concluded that the PIB injection method of local anesthetic produces greater pressure and spreads better around the nerve root than the continuous injection method. The increased pressure of injecting local anesthetic with the PIB method probably compensates for the distance between the catheter's orifice and the target nerve, making it easier to reach the nerve fascicles, reducing local anesthetic consumption, and providing effective analgesia. A cadaveric tissue model proved that the intermittent volume spreads better in the epidural space [15, 16], but this was not fully demonstrated in a peripheral tissue model. The increased distribution of local anesthetic with a properly placed perineural catheter should produce a more complete sensory block. This enhanced sensory block may be accompanied by an increased motor block [17].

Few studies have investigated the effect of local anesthetic injection methods on pain improvement and adverse events during CIBPB. A study reported that 0.2% ropivacaine was administered at 1 h intervals of 5 mL/h with a bolus dose in CIBPB; the PIB method showed a similar analgesic efficacy and a significant reduction in the total consumption of local anesthetic and incidence of the motor blockade [18]. In another study, PIB of local anesthetic combined with PRN boluses did not reduce local anesthetic consumption or rescue analgesia compared to continuous infusion combined with PRN boluses [19].

In our study, we expected that local anesthetic administration using the PIB method would also show a better analgesic effect in CIBPB. However, regarding differences in pain control, the PIB method did not show better results than the continuous infusion method. Rather, the initial 24 h bolus usage increased, resulting in more local anesthetic consumption and motor blockades. We also expected that the PIB injection of local anesthetics would have a greater effect than the continuous injection of small amounts. However, the effect of pain control was not greater than that of local anesthetics. Our study injected 1.1 mL/h using the continuous infusion method to match the amount of local anesthetic infused in the two groups, and 4 mL was injected every 4 h using the PIB method. In the PIB method, 0.1 mL/h was injected so that the permanent catheter was not occluded, and as a result, 4.4 mL of local anesthetic was injected for 4 h. This was done to consider the elimination half-life of 0.2% ropivacaine and to some extent, the risk of systemic local anesthetic toxicity that may occur when injected in excess. In a previous study, the minimum effective volume (MEV) of 0.5% bupivacaine with epinephrine in 90% of patients was 0.95 mL, and a 2.34 mL of MEV_90_ reported that there was little pain for 6 h, proving that very low volumes can be successfully used with ultrasound-guided CIBPB [20]. In our previous study [10], a 4 mL bolus only showed the same analgesic effect as the group injected with continuous infusion. Therefore, we thought that the PIB method would be more efficacious in controlling pain and injecting local anesthetics.

Our findings show that injecting small amounts of local anesthetics at long intervals of 4 h was less effective than the continuous infusion method. Therefore, the patient wanted more bolus injections. Based on these results, it will be necessary to investigate the effect of administering local anesthetics using the PIB method even in various surgeries that can perform continuous peripheral nerve blocks. It will also be necessary to explore the optimal bolus dose or intermittent interval in the PIB method for each continuous peripheral nerve block location according to various surgeries.

A limitation of our study is the small sample size; thus, it is necessary to conduct a further study with a larger sample. Some parameters, such as the total dose of local anesthetics, frequency, and dose of bolus injection, and others are expected to be statically meaningful with large sample sizes. Since interscalene brachial plexus catheter insertion is a procedure with high failure rates and adverse events, there is a need for a study that assesses these negative factors. As another limitation, since the pressure applied to the catheter is different in the continuous infusion method and in the PIB method, the position of the catheter tip may change, which will affect the results. It was necessary to check whether the catheter tip was located in the nerve trunk of the targeted brachial plexus. However, we could not confirm the position of the catheter tip during the follow-up. To confirm the position of the catheter tip by ultrasound, a sticky dressing had to be removed, and there was a high risk of pull-out of the perineural catheter during this process.

In conclusion, the PIB method in CIBPB after arthroscopic shoulder surgery provided a similar analgesic effect, with a higher bolus injection dose of local anesthetic and increased motor blockade than the continuous infusion method.

## Figures and Tables

**Table 1:** Demographic data.

Characteristic	Group A (*n* = 32)	Group B (*n* = 32)
Sex (M/F)	14/18	14/18
ASA physical status (I/II)	9/23	10/22
Age (years)	57.6 ± 13.0	59.1 ± 10.0
Height (cm)	162.2 ± 10.8	160.5 ± 8.1
Weight (kg)	66.0 ± 14.0	67.2 ± 8.7
Anesthesia time (hours)	1.6 ± 0.4	1.7 ± 0.3

All measured values are presented as the mean ± standard deviation or number of patients. ASA: American Society of Anesthesiologists.

**Table 2:** Visual analog scale (VAS) scores and incidence of rescue analgesic at 0, 24, and 48 h after surgery.

Time (h)		Group A (*n* = 28)	Group B (*n* = 29)	*p* value
0	VAS	21.8 ± 14.7	20.7 ± 15.3	0.784
	Rescue analgesic	4 (14.3)	6 (20.7)	0.730

24	VAS	37.1 ± 15.4	40.7 ± 21.0	0.472
	Rescue analgesic	14 (50.0)	13 (44.8)	0.793

48	VAS	27.9 ± 14.0	29.3 ± 15.3	0.710
	Rescue analgesic	7 (25.0)	5 (17.2)	0.530

All measured values are presented as the mean ± standard deviation or number of patients (%). ^*∗*^*p* < 0.05 0 h: immediately after admission to the PACU.

**Table 3:** Motor block scores using a modified Bromage scale (MBS) analgesic at 0, 24, and 48 h after the surgery.

Time (h)	MBS	Group A (*n* = 28)	Group B (*n* = 29)	*p* value
0	Grade 0	18 (64.3)	12 (41.4)	0.221
	Grade 1	9 (32.1)	15 (51.7)	
	Grade 2	1 (3.6)	2 (6.9)	

24	Grade 0	24 (85.7)	17 (58.6)	0.038^*∗*^
	Grade 1	4 (14.3)	12 (41.4)	

48	Grade 0	26 (92.9)	27 (93.1)	1.000
	Grade 1	2 (7.1)	2 (6.9)	

All measured values are presented as the number of patients (%). ^*∗*^*p* < 0.05 0 h: immediately after admission to the PACU. Grade 0: able to raise the extended arm to 90° for 2 s. Grade 1: able to flex the elbow and move the fingers but unable to raise the extended arm. Grade 2: unable to flex the elbow but able to move the fingers. Grade 3: unable to move the arm, elbow, or fingers.

**Table 4:** Administration of local anesthetic through the patient-controlled device.

Administration of local anesthetic through the patient-controlled device	Group A (*n* = 28)	Group B (*n* = 29)	*p* value
Total dose (mL)	92.0 ± 35.5	108.3 ± 38.6	0.104
*Bolus injection dose (mL)*			
Bolus injection dose at 24 h (mL)	15.7 ± 7.3	20.0 ± 8.7	0.047^*∗*^
Bolus injection dose at 48 h (mL)	12.6 ± 4.9	13.7 ± 6.7	0.468
*Frequency of using bolus at 24 and 48 h*			
At 24 h	1.8 ± 1.9	3.0 ± 2.4	0.034^*∗*^
At 48 h	1.0 ± 1.2	1.2 ± 1.6	0.589

All measured values are presented as the mean ± standard deviation or number of patients (%). ^*∗*^*p* < 0.05.

**Table 5:** Patient satisfaction with postoperative pain management.

Patient satisfaction	Group A (*n* = 28)	Group B (*n* = 29)	*p* value
5 = very satisfied	4 (14.3)	7 (24.1)	0.457
4 = satisfied	12 (42.9)	12 (41.4)	
3 = neutral	8 (28.6)	5 (17.2)	
2 = dissatisfied	4 (14.3)	3 (10.3)	
1 = very dissatisfied	0 (0.0)	2 (3.5)	

All measured values are presented as the number of patients (%). ^*∗*^*p* < 0.05.

**Table 6:** Incidences of adverse events.

Adverse events	Group A (*n* = 28)	Group B (*n* = 29)	*p* value
Postoperative nausea and vomiting (PONV)	3 (10.7)	3 (10.3)	0.648
Dizziness	2 (7.1)	1 (3.4)	0.487
Hypotension	0 (0.0)	0 (0.0)	1.000
Paresthesia	6 (21.4)	12 (41.4)	0.091
Urinary retention	0 (0.0)	2 (6.9)	0.254

All measured values are presented as the number of patients (%). ^*∗*^*p* < 0.05.

## Data Availability

The data are available from the authors upon request, through a data access committee, institutional review board, and reviewers. If anyone requires the data from this study, please do not hesitate to contact the corresponding author (Gyeong-Jo Byeon, e-mail: byeongj@pusan.ac.kr).
